# Investigation of Rapid Non-Isothermal Crystallization Kinetics of Polyamide 66 Using a Fast-Scanning Chip-Based DSC

**DOI:** 10.3390/s26092680

**Published:** 2026-04-25

**Authors:** Shaokui Tan, Ming Li, Zechun Li, Jun Yan, Zhihao Zhang, Pengcheng Xu, Peide Wu, Xinxin Li

**Affiliations:** 1 College of Chemistry and Materials Science, Shanghai Normal University, Shanghai 200234, China; 2 State Key Laboratory of Transducer Technology, Shanghai Institute of Microsystem and Information Technology, Chinese Academy of Sciences, Shanghai 200050, China; liming01@mail.sim.ac.cn (M.L.); xpc@mail.sim.ac.cn (P.X.); 3 University of Chinese Academy of Sciences, Beijing 101408, China; 4 School of Opto-Electronic Engineering, Guilin University of Electronic Technology, Guilin 541004, China

**Keywords:** non-isothermal crystallization, kinetic, fast-scanning, chip-based DSC, MEMS thermopile

## Abstract

Understanding the rapid non-isothermal crystallization behavior of polymers is crucial for tailoring and optimizing their performance. However, conventional techniques are limited in achieving rapid heating and cooling rates, which hinders in-depth investigation of the crystallization kinetics of fast-crystallizing polymers. In this study, a high-scan-rate MEMS thermopile DSC chip is employed to systematically investigate the non-isothermal crystallization kinetics of polyamide 66 (PA66) under rapid temperature variations. The results show that PA66 forms a lamellar α phase under slow cooling (1 °C/s) and a cauliflower-like γ phase under rapid cooling (300 °C/s), and becomes completely amorphous under ultrafast cooling (quenching). Furthermore, the technique enables quantitative analysis of the cold crystallization kinetics of fully amorphous PA66 during rapid heating. The results indicate that PA66 exhibits a higher apparent activation energy for homogeneous nucleation cold crystallization at low heating rates (≤10 °C/s), reaching 172.3 kJ·mol^−1^, which is approximately 3.2 times that at high heating rates (≥25 °C/s). The results of this study demonstrate that the developed fast-scanning chip-based DSC provides a powerful tool for analyzing the processing heating and cooling rate conditions of rapidly crystallizing polymers.

## 1. Introduction

Polymeric materials, owing to their tunable chemical structures and diverse physical properties [1,2,3,4], have become indispensable foundational materials in modern industry. The crystallization behavior of polymers directly governs their performance. Higher crystallinity leads to more closely packed molecular chains and stronger intermolecular interactions, which generally enhance mechanical strength [5,6], thermal stability [7], and barrier properties [8,9], but often reduce optical transparency [10]. Therefore, controlling the crystallization behavior is essential for achieving high-performance polymeric materials. In particular, fast-crystallizing polymers, characterized by rapid crystallization rates and short processing times during injection molding, extrusion, and forming, are of great importance for efficient large-scale production. To precisely regulate these rapid crystallization processes and achieve target properties, a thorough understanding of their crystallization kinetics is required. However, elucidation of the kinetics of fast-crystallizing polymers strongly depends on experimental platforms capable of rapid heating and cooling. Only under accurately controlled temperature variation rates can the transient structural evolution during crystallization be captured and the corresponding transient kinetic products be obtained, thereby providing a reliable basis for optimizing the temperature program in the processing of high-performance polymers.

In polymer crystallization studies, a variety of characterization techniques are widely employed to probe structures at different length scales. For example, polarized optical microscopy is used to observe the morphology and size of spherulites [11,12,13], enabling analysis of crystallization morphology and growth processes. Wide-angle X-ray diffraction characterizes crystal structures [14,15,16], while atomic force microscopy is suitable for nanoscale morphological analysis of surfaces and interfaces [17,18]. However, polymer processing and application are typically accompanied by temperature variations, necessitating dynamic investigation through thermal analysis techniques. Among these, differential scanning calorimetry (DSC) has long served as an indispensable tool in this field [19,20]. DSC enables precise measurement of heat flow during crystallization and melting, providing key thermodynamic parameters, including crystallization/melting temperatures, enthalpies, and crystallinity [21,22,23]. In addition, by varying heating and cooling rates, DSC has been extensively used to investigate crystallization kinetics, offering critical insights into nucleation and growth mechanisms [24,25,26]. Nevertheless, conventional DSC, based on a furnace–crucible–thermocouple configuration, suffers from inherent thermal inertia, which limits its maximum heating and cooling rates to the order of 100 °C/min. This constraint makes it inadequate for studying the rapid non-isothermal crystallization kinetics of polymers. With the rapid development of MEMS technology, thermocouples and heating elements can be integrated onto a single miniaturized chip, enabling the fabrication of MEMS-based DSC with ultrafast heating and cooling capabilities [27,28,29], thereby filling a critical gap in materials research within this field. Although Flash DSC has been progressively applied to the investigation of polymer crystallization behavior, existing studies have predominantly focused on isothermal crystallization processes [30,31,32], with comparatively limited attention given to non-isothermal crystallization.

In this work, the non-isothermal rapid crystallization behavior of nylon 66 (PA66) is systematically investigated by using a MEMS thermopile DSC chip, and the apparent activation energy associated with homogeneous nucleation cold crystallization is further analyzed. Owing to the ultralow heat capacity and thermally insulated structural design achieved by MEMS technology, the chip provides a high signal-to-noise ratio and sensitivity, while simultaneously offering excellent heating and cooling performance. Using this chip-based DSC, the crystallization kinetics of a typical fast-crystallizing polymer, PA66, were systematically investigated. The results show that variations in cooling rate regulate the crystallization morphology of PA66, thereby influencing the resulting crystal structure and crystallinity. Furthermore, the kinetics of homogeneous nucleation cold crystallization of PA66 at different heating rates were quantitatively analyzed, revealing that the apparent activation energy at low heating rates is approximately 3.2 times that at high heating rates. The application of this fast-scanning chip-based DSC to the study of crystallization kinetics provides important implications for the control and optimization of polymer processing.

## 2. Materials and Methods

### 2.1. Materials

PA66 was purchased from Sigma-Aldrich (Shanghai) Trading Co., Ltd., Shanghai, China (CAS No. 32131-17-2, product No. 429171). The moisture content was <0.06%, and the relative viscosity was 200–300.

### 2.2. Sample Loading

A small thin slice was cut from the purchased PA66 polymer granules using a clean blade. The slice was then picked up with a fine microprobe under electrostatic attraction and, with the aid of a microscope, carefully positioned at the center of the sensing thermopile on the chip. The sample is 37 μm thick and weighs approximately 0.418 μg (Figure S1).

### 2.3. Testing System

The MEMS thermopile DSC chip loaded with the PA66 sample was installed in a dedicated sealed chamber. The inert atmosphere inside the chamber was controlled by a LoC-GDS 4000 intelligent gas mixing system (High-End MEMS Technology Co., Ltd., Xiamen, China), using high-purity Ar as the protective gas. Prior to formal measurements, the chamber was purged with Ar at a flow rate of 200 mL/min for 10 min to ensure complete removal of air and moisture. During subsequent DSC measurements, the gas flow rate was maintained at 100 mL/min. To optimize the thermal contact between the sample and the sensing area of the chip, the sample was slowly heated to 360 °C (above the melting point of PA66) at 1 °C/s and briefly held prior to non-isothermal crystallization measurements, ensuring intimate thermal contact with the chip surface.

### 2.4. Morphological Characterization

Owing to the miniaturized and open structure of the MEMS thermopile DSC chip, characterization of PA66 samples cooled to room temperature at different rates using atomic force microscopy (AFM, Dimension Icon, Bruker, Karlsruhe, Germany) is straightforward. The chip can be directly placed on the sample stage, and the AFM probe positioned above the center of the thermopile, allowing easy acquisition of surface morphology for samples subjected to different cooling treatments. Imaging was performed in tapping mode with a working frequency of 300 kHz. AFM image processing and analysis were conducted using the NanoScope Analysis 3.0 software provided by Bruker.

## 3. Results and Discussion

### 3.1. Chip DSC Testing System

**Figure 1:**
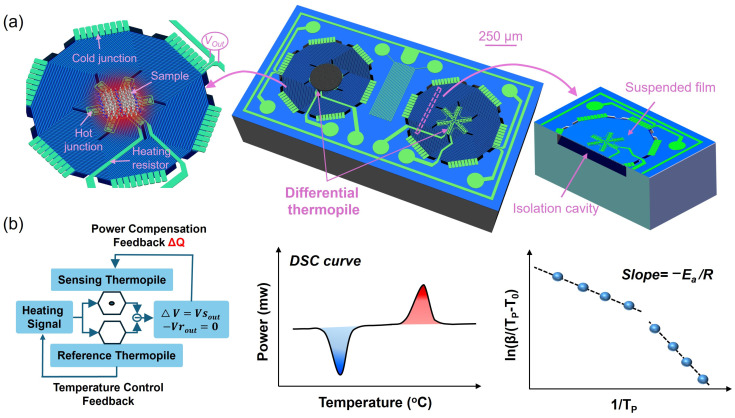
Structure of MEMS thermopile DSC chip and Principles of testing polymer kinetic behavior. (**a**) Schematic structure of the MEMS thermopile DSC chip; (**b**) Principle of polymer kinetic measurements using the MEMS thermopile DSC chip.

The structural design of the MEMS thermopile DSC chip is shown in Figure 1a. The specific manufacturing process for this chip is presented in Figure S2. The overall chip size is 2 mm × 1 mm, and it consists of two thermopiles. The PA66 sample is placed at the central region of the sensing thermopile, while the other thermopile is kept empty as a reference. The MEMS thermopile employs high-sensitivity N/P-type single-crystal silicon as the thermocouple material. Through a microhole-interetching-sealing (MIS) process, 54 pairs of N/P-type single-crystal silicon thermocouples are integrated beneath the silicon nitride (SiN_x_) thin film, forming a thermally insulated cavity structure. The SiN_x_ thin film has a thickness of 1 μm, a suspended membrane diameter of 640 μm, and a thermal conductivity of 4 W/(m·K), while the cavity depth is 40 μm. The hot junctions are distributed at the center of the suspended SiN_x_ membrane, with a central diameter of 240 μm, and the metal heater is arranged around the hot junctions (Figure S3). The cold junctions are positioned along the edge of the membrane and are connected to the silicon substrate, thereby maintaining thermal equilibrium with the ambient temperature. Based on the Seebeck effect, the output voltage of the thermopile is proportional to the temperature difference between the hot and cold junctions [33], enabling the thermopile to control and measure temperature. Owing to the thermally insulated cavity structure, the chip exhibits ultralow heat capacity and excellent thermal isolation, endowing it with ultrafast heating and cooling capabilities.

The principle of polymer kinetic measurements using the MEMS thermopile DSC chip is illustrated in Figure 1b. The temperatures of the two thermopiles can be controlled by adjusting the heating voltage. During programmed heating and cooling, a closed-loop temperature control system dynamically applies power compensation to the sensing thermopile to maintain the same temperature as the reference thermopile. Under these conditions, power-compensated DSC measurements can be achieved by monitoring the power difference between the sensing and reference thermopiles. Owing to the ultrafast dynamic thermal response of the MEMS thermopile DSC chip, fully amorphous PA66 can be readily obtained through rapid cooling quenching. *T_O_* investigate the kinetics of homogeneous nucleation crystallization, DSC curves were measured at different heating rates, and key kinetic parameters, including the onset temperature (*T_O_*), peak temperature (*T_P_*), and corresponding heating rates, were extracted for the homogeneous nucleation cold crystallization process. On this basis, the Augis–Bennett (A-B) model was employed to quantitatively determine the apparent activation energy of homogeneous nucleation cold crystallization across different heating rate regimes.

### 3.2. Chip Performance Characterization

**Figure 2:**
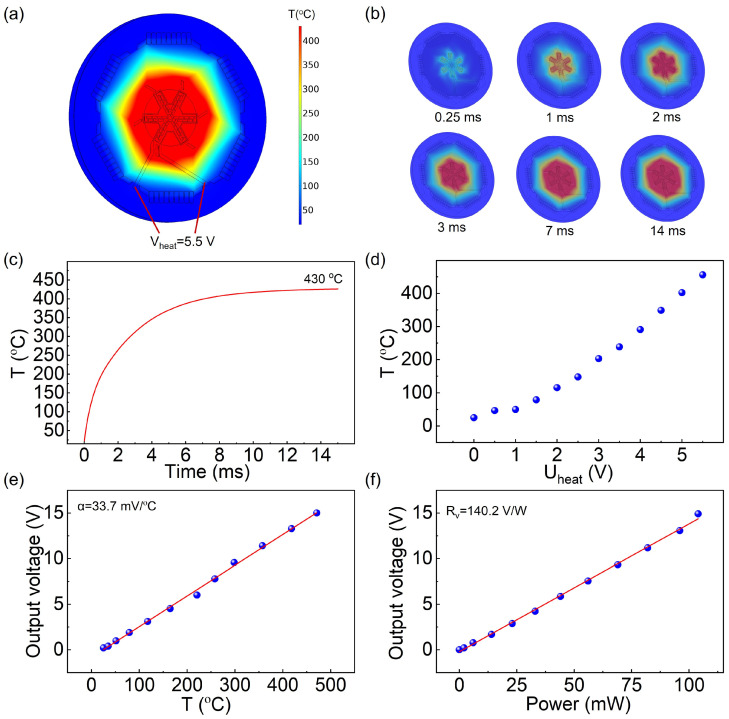
Performance characterization of the MEMS DSC chip. (**a**) COMSOL simulation of the temperature distribution at a heating voltage of 5.5 V; (**b**) Transient COMSOL simulation of the thermopile temperature response to a step change; (**c**) COMSOL simulation curves for temperature step changes in a thermoelectric stack; (**d**) Measured relationship between thermopile heating voltage and temperature; (**e**) Temperature response of the thermopile output voltage; (**f**) Power response of the thermopile output voltage.

In this study, COMSOL Multiphysics 6.3 was employed to simulate the temperature distribution of the MEMS thermopile DSC chip. The detailed simulation input parameters are provided in Table S1 of the Supporting Information. As shown in Figure 2a and Figure S4, the temperature distribution at a heating voltage demonstrates the uniformity of temperature in the central sensing region of the thermopile, with a temperature deviation of less than 5%. Ensuring that all microdomains within the sample undergo transformation within a strictly uniform temperature field effectively eliminates peak broadening induced by local temperature gradients. Consequently, the measured heat flow curves of the material more closely reflect the intrinsic kinetic behavior, thereby improving the accuracy of the extracted kinetic parameters. As illustrated in Figure 2b, transient simulations were conducted to evaluate the dynamic thermal response of the thermopile. When the heating voltage is 5.5 V, the temperature reaches an equilibrium value of 430 °C within only 14 ms (Figure 2c). The step response characteristics of the chip during heating and cooling were evaluated. The measured time constants for heating and cooling are 2.0 ms and 2.4 ms, respectively, while the maximum heating and cooling rates reach 1.6 × 10^5^ °C/s and 1.3 × 10^5^ °C/s, respectively (Figure S5). Such an ultrafast thermal response enables precise temperature control during rapid heating and cooling processes, which is critical for characterizing materials with fast thermal transitions.

As shown in Figure 2d, an infrared camera (Model: TiX560) manufactured by Fluke was used to observe the temperature in the central region of the thermopile, evaluating the relationship between heating voltage and temperature. At a heating voltage of 5.5 V, the measured temperature is 455 °C, which agrees well with the simulation results, confirming their accuracy. As presented in Figure 2e, the temperature response of the thermopile output voltage was tested, showing a good linear relationship between output voltage and temperature, with a sensitivity of 33.7 mV/°C. In addition, owing to its thin-film structure, the thermopile exhibits a power responsivity of up to 140.2 V/W (Figure 2f), enabling highly sensitive detection of the weak heat flow associated with sample phase transitions. Compared with the commercial UFS1 chip from Mettler Toledo [29], the MEMS thermopile DSC chip employed in this study exhibits a higher temperature response and power response, as well as a smaller cooling time constant (Table S2). Furthermore, the temperature accuracy of the chip-based DSC was characterized using metallic indium and tin. The melting points of indium and tin were measured at a heating rate of 100 °C/s, yielding values of 156.52 °C and 232.05 °C, respectively. These results are in excellent agreement with the standard values (156.51 °C and 231.89 °C), thereby confirming the high accuracy of temperature measurement of the DSC chip (Figure S6). These results demonstrate that the MEMS thermopile DSC chip possesses excellent performance, enabling the analysis of rapid crystallization kinetics in polymers.

### 3.3. Non-Isothermal Crystallization Behavior of PA66

**Figure 3:**
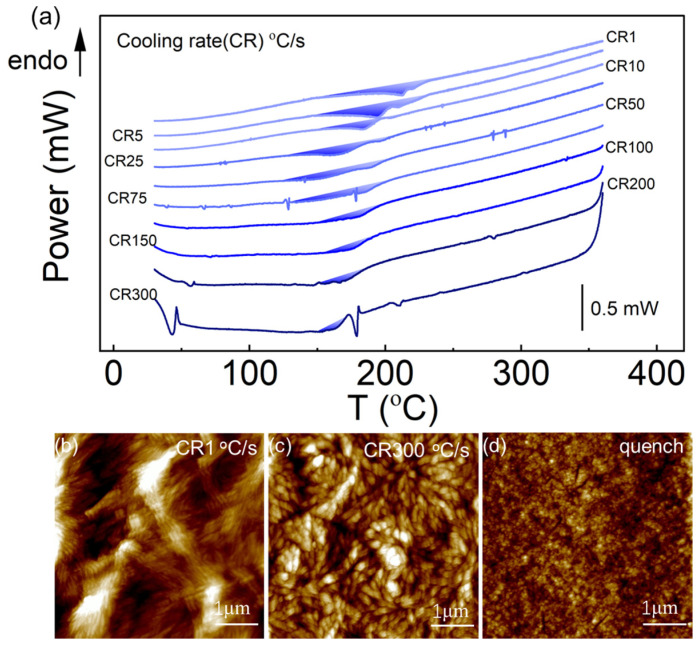
Effect of cooling rate on the melt crystallization of PA66. (**a**) DSC cooling curves at different cooling rates, the upward arrow indicates the endothermic (endo) direction; (**b**–**d**) AFM images of PA66 cooled at 1 °C/s, 300 °C/s, and by quenching, respectively.

Figure 3a shows the DSC cooling curves of PA66 at different cooling rates, where the upward arrow indicates the endothermic (endo) direction. The same convention applies to all DSC curves presented below. An exothermic crystallization peak is observed in the DSC curves at cooling rates of 1–300 °C/s, indicating that crystallization occurs during the cooling process. The sharp peak near 180 °C in the DSC curve at a cooling rate of 300 °C/s is attributed to system noise. To further elucidate the morphology and structural characteristics of PA66 under different cooling conditions, AFM was employed to characterize the surface morphology of samples cooled to room temperature. Compared with previously reported results [31,32], as shown in Figure 3b, the sample cooled at 1 °C/s exhibits a typical lamellar morphology, consistent with the characteristics of the α crystalline phase. As shown in Figure 3c, the sample subjected to rapid cooling at 300 °C/s displays a cauliflower-like morphology, which is attributed to the γ crystalline phase. Furthermore, to obtain PA66 samples subjected to ultrafast cooling, the MEMS thermopile DSC chip can be used to heat the sample to the target temperature, after which the heating voltage is immediately set to zero. Owing to the good dynamic thermal response of the chip, the sample can be rapidly cooled to room temperature within an extremely short time, enabling the acquisition of samples subjected to quenching treatment. The quenching cooling rate of the sample is estimated to be approximately 4177 °C/s, and the instantaneous cooling rate near 150 °C can reach about 1.03 × 10^4^ °C/s (Figure S7). As shown in Figure 3d, no distinct crystalline phase was observed in the quenched samples, indicating a fully amorphous state. In addition, under identical thermal treatment conditions, micro-infrared spectroscopy measurements were performed on PA66 samples cooled at 1 °C/s and 300 °C/s, as well as on samples quenched to room temperature. The obtained results are consistent with the observations from atomic force microscopy (AFM) (Figure S8) [34,35,36,37]. This demonstrates that the cooling rate has a significant impact on the crystal structure and degree of crystallinity of PA66.

**Figure 4:**
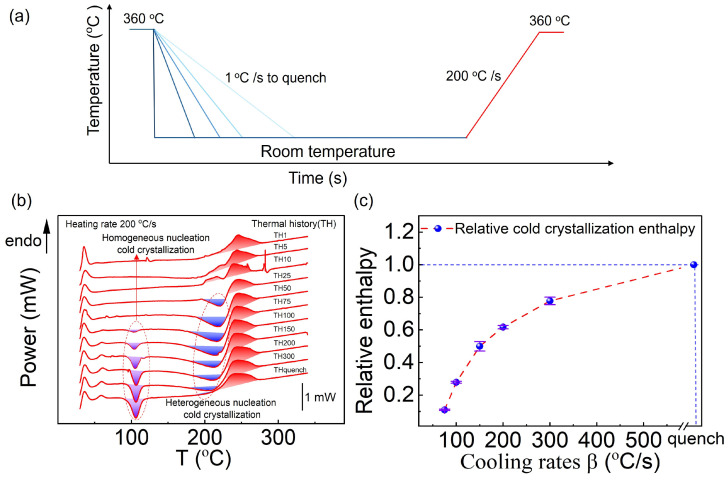
Effect of prior cooling rate on cold crystallization kinetics during reheating. (**a**) Schematic of the temperature program used in this study. The blue and red curves represent the programmed cooling and heating processes, respectively; (**b**) DSC curves of PA66 with different thermal histories, reheated at a fixed rate of 200 °C/s (TH1 corresponds to a prior cooling rate of 1 °C/s, with subsequent labels following the same order); (**c**) Relationship between cold crystallization enthalpy and prior cooling rate.

To investigate the influence of cooling rate on the subsequent cold crystallization behavior of PA66, thermal analysis was conducted following the procedure shown in Figure 4a. First, the sample was heated to 360 °C to erase its thermal history. It was then cooled to room temperature under cooling rates ranging from 1 to 300 °C/s, as well as under quenching conditions. Subsequently, the sample was reheated at a fixed rate of 200 °C/s to examine its cold crystallization kinetics.

The prior cooling rate can affect the composition and structure of PA66, which in turn influences its subsequent cold crystallization behavior. During reheating, cold crystallization can be classified into heterogeneous nucleation and homogeneous nucleation. Heterogeneous nucleation cold crystallization occurs when external surfaces, impurities, substrates, or residuals from previous crystals act as nucleation sites to induce crystal growth [38,39]. In contrast, homogeneous nucleation cold crystallization arises from the spontaneous reorganization of molecular chain segments to form ordered microdomains, generating nuclei intrinsically [40,41]. Figure 4b shows the DSC curve obtained at a heating rate of 200 °C/s. Compared with previously reported results [42], a relatively lower heating rate was employed in this study in order to observe the exothermic behavior associated with heterogeneous nucleation cold crystallization. It has been found that the degree of heterogeneous nucleation cold crystallization during the subsequent heating process exhibits a trend of first increasing and then decreasing with increasing prior cooling rate. This phenomenon can be explained from the perspective of the crystal nucleus origin. The nuclei responsible for heterogeneous nucleation cold crystallization are predominantly derived from imperfect microcrystals formed during the preceding cooling stage, as well as from spherulites generated via homogeneous nucleation cold crystallization during the heating stage. The crystallization behavior of polymers is primarily governed by the combined effects of nucleation and diffusion processes [43]. Therefore, at the initial stage of increasing cooling rate, the crystallization undercooling of the polymer gradually increases, and crystal growth becomes insufficient during cooling, leading to the formation of a large number of microcrystals. These microcrystals provide abundant nucleation sites for subsequent heterogeneous nucleation, thereby enhancing the degree of heterogeneous nucleation cold crystallization. However, at extremely high cooling rates, polymer chains do not have sufficient time to aggregate and form an adequate number of nuclei, which in turn leads to a decrease in the degree of heterogeneous nucleation cold crystallization [44,45].

**Figure 5:**
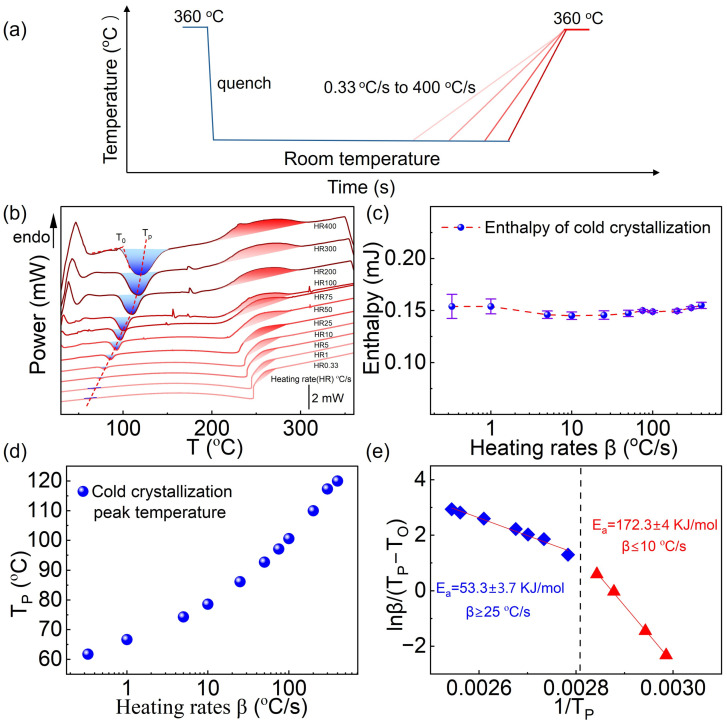
Study of homogeneous nucleation cold crystallization kinetics of PA66. (**a**) Schematic of the quenching–reheating test program. The blue and red curves represent the programmed cooling and heating processes, respectively; (**b**) DSC curves at different heating rates. The dashed line shows the variation trend of T_P_ on DSC curves at different heating rates; (**c**) Variation in cold crystallization enthalpy with heating rate; (**d**) Variation in cold crystallization peak temperature with heating rate; (**e**) Activation energy analysis based on the Augis–Bennett model. The blue and red dots denote the 1/T_P_ values for activation energy calculation at high and low heating rates, respectively.

Further analysis of the DSC curves revealed that homogeneous nucleation cold crystallization begins to occur when the cooling rate exceeds 75 °C/s. In addition, the enthalpy of homogeneous nucleation cold crystallization gradually increases with increasing cooling rate and approaches saturation at 300 °C/s (Figure 5c). When PA66 is fully amorphous after quenching, the enthalpy of homogeneous nucleation cold crystallization reaches its maximum. These results enable a quantitative determination of the boundary conditions for the occurrence of homogeneous nucleation cold crystallization, thereby providing a theoretical basis for the optimization of polymer processing conditions.

### 3.4. Non-Isothermal Cold Crystallization Kinetics

To investigate the effect of heating rate on the cold crystallization behavior of fully amorphous PA66, the temperature program shown in Figure 5a was employed. First, PA66 was held at 360 °C for 1 s to erase its thermal history. The sample was then rapidly cooled (quenched) to obtain a fully amorphous state, followed by reheating at various rates ranging from 0.33 to 400 °C/s.

The DSC curves at different heating rates are shown in Figure 5b. By integrating these curves over time, the corresponding cold crystallization enthalpies were obtained (Figure 5c). The results indicate that the enthalpy of homogeneous nucleation cold crystallization remains largely unchanged with increasing heating rate, suggesting that homogeneous nucleation behavior is independent of the heating rate, providing a basis for studying intrinsic crystallization kinetics.

Past studies on polymer crystallization kinetics have frequently employed the Avrami kinetic model, one of the most typical and widely used theoretical frameworks [46]. The kinetic expression of the Avrami model is given by:

(1) $$x=1-exp[-\left(kt{)}^{n}\right]$$

where *x* represents the fraction of material transformed at time *t*, *n* is a dimensionless exponent. The rate constant *k* follows the Arrhenius relationship, which describes its dependence on temperature.

(2) $$k=\nu \;exp-\frac{E_{a}}{RT}$$

where *ν* is the frequency factor, *E_a_* is the activation energy, *R* is the gas constant, and *T* is the temperature in Kelvin. The Avrami model is mathematically simple yet provides a bridge between macroscopic observations and microscopic mechanisms. Importantly, the exponent *n* is not merely a parameter; it directly relates to the microscopic nucleation mechanism of polymer crystallization. However, the model is limited to describing isothermal crystallization processes.

In practical polymer processing, materials often undergo continuous heating and cooling, i.e., non-isothermal conditions. Therefore, there is a critical need to establish kinetic models capable of accurately describing non-isothermal crystallization, which better reflect real-world polymer production and processing behavior. Among various models developed to describe non-isothermal phase transition kinetics, the Kissinger model is one of the most classical and widely applied [47]. Its kinetic expression is:

(3) $$\ln\frac{\beta }{T_{P}^{2}}=C_{KS}-\frac{E_{a}}{RT_{P}}$$

where *β* is the heating rate, *T_P_* is the peak temperature observed in DSC during the phase transition, and *C_KS_* is the Kissinger constant. Nevertheless, the Kissinger model has limitations: it requires linear heating rates, the phase transition must follow single-step kinetics, and the phase transition kinetics must be independent of the heating rate.

The Augis–Bennett (A-B) model integrates the advantages of the two aforementioned models [48], effectively modifying the Avrami model through the Kissinger approach, thereby extending the isothermal process to non-isothermal conditions. This modification allows the model to account for the influence of nucleation factors during the crystallization process without requiring a predetermined reaction order. Using this model, the apparent activation energy of fully amorphous PA66 can be quantitatively analyzed under different heating rates. The model is expressed as:

(4) $$\ln\left(\frac{\beta }{T_{P}-T_{O}}\right)=C-\frac{E_{a}}{RT_{P}}$$

where *T_O_* is the onset temperature of homogeneous nucleation and cold crystallization, and *C* is a constant.

The *T_P_* value in Figure 5b was marked with a dashed line, and the corresponding data were processed as shown in Figure 5d, the homogeneous nucleation cold crystallization peak temperature of fully amorphous PA66 increases with the heating rate, consistent with the fundamental principles of non-isothermal phase transition kinetics. As illustrated in Figure 5e, linear fitting of *ln*(*β*/(*T_P_ − T_O_*)) versus *1*/*T_P_* allows the calculation of the apparent activation energy for cold crystallization via homogeneous nucleation. At low heating rates (≤10 °C/s), the apparent activation energy is determined to be 172.3 kJ·mol^−1^, whereas at high heating rates (≥25 °C/s), it decreases to 53.3 kJ·mol^−1^. The observed decrease in apparent activation energy is in agreement with previously reported isothermal studies on homogeneous nucleation kinetics. It indicates that, under high heating rates, the energy barrier for molecular segment rearrangement during non-isothermal homogeneous nucleation and cold crystallization is reduced, resulting in a faster crystallization process. This highlights the relationship between the homogeneous nucleation rate and the heating conditions. Further investigation revealed that the heterogeneous nucleation and cold crystallization process is gradually suppressed with increasing heating rate, and completely disappears at a rate of 400 °C/s. At this point, the original change in the baseline slope around 250 °C in the DSC heating curve also disappears accordingly (Figure S9). These findings not only deepen the understanding of the rapid crystallization behavior of PA66 but also provide essential kinetic insight for tailoring processing conditions to achieve functional modifications.

## 4. Conclusions

This study systematically investigated the non-isothermal crystallization kinetics of PA66 using a high-scan MEMS thermopile DSC chip. The results show that PA66 forms an α-phase crystalline structure at low cooling rates and a γ-phase structure at high cooling rates, and remains completely amorphous under ultrafast cooling (quench). Furthermore, different cooling rates influence the subsequent cold crystallization behavior upon reheating, allowing the critical conditions for homogeneous nucleation cold crystallization to be determined. Furthermore, this study quantitatively analyzes the variation in the kinetic energy barrier associated with homogeneous nucleation cold crystallization. The apparent activation energy at low heating rates is 172.3 kJ·mol^−1^, which is significantly higher than that at high heating rates (53.3 kJ·mol^−1^). These results indicate that the heating rate has a pronounced effect on homogeneous nucleation cold crystallization. The fast-scanning chip-based DSC provides a novel platform for elucidating the rapid crystallization kinetics of fast-crystallizing polymers and offers important guidance for optimizing polymer processing conditions.

## Data Availability

The data presented in this study are available on request from the corresponding author due to (specify the reason for the restriction).

## Supplementary Materials

The following supporting information can be downloaded at: https://www.mdpi.com/article/10.3390/s26092680/s1, Figure S1: Optical images of the PA66-loaded sample; Figure S2: MEMS thermopile DSC chip fabrication process; Figure S3: Optical microscopy image of the MEMS thermopile DSC chip; Figure S4: Temperature distribution maps at heating voltages of 2 V, 3 V, and 4 V; Figure S5: Step response of the MEMS thermopile DSC chip under unloaded conditions. (a) Heating step response curve; (b) Cooling step response curve; Figure S6: DSC melting curves of indium and tin measured using the MEMS thermopile DSC chip; Figure S7: Quenching cooling rate measurement of the chip after loading the sample: (a) quenching cooling curve and its first derivative; (b) evaluation of the average cooling rate during the quenching process; Figure S8: Infrared absorption spectra of samples subjected to different cooling conditions; Figure S9: Integration analysis of heterogeneous nucleation cold crystallization enthalpy of quenched PA66 at different heating rates. Table S1: Input parameters for the COMSOL finite element simulation; Table S2: Comparison of parameters between the MEMS thermopile DSC chip and the commercial chip UFS1.
