# Supplementary material for: Investigation of Rapid Non-Isothermal Crystallization Kinetics of Polyamide 66 Using a Fast-Scanning Chip-Based DSC
Source: Sensors (Basel). 2026 Apr 25;26(9):2680. doi: 10.3390/s26092680 (PMC13166010; doi:10.3390/s26092680)
Supplement: Supplementary file 1 [file sensors-26-02680-s001.zip › sensors-4258175-supplementary.pdf]

Supporting Information

Investigation of Rapid Non-Isothermal Crystallization Kinetics of Polyamide 66 Using a Fast-Scanning Chip-Based DSC

Shaokui Tan[1, 2], Ming Li[2, 3], Zechun Li[2, 3], Jun Yan[2, 4], Zhihao Zhang[1, 2], Pengcheng Xu[2, 3], Peide Wu[2*] and Xinxin Li[2, 3*]

1 College of Chemistry and Materials Science, Shanghai Normal University, Shanghai 200234, China

tanshaokui@mail.sim.ac.cn (S.T.); zhangzhihao@mail.sim.ac.cn (Z.Z.)

2 State Key Laboratory of Transducer Technology, Shanghai Institute of Microsystem and Information Technology, Chinese Academy of Sciences, Shanghai 200050, China

zechunli@mail.sim.ac.cn (Z.L.); qq630488014@163.com (J.Y.); xpc@mail.sim.ac.cn (P.X.)

3 University of Chinese Academy of Sciences, Beijing 101408, China

4 School of Opto-Electronic Engineering, Guilin University of Electronic Technology, Guilin 541004, China

* Correspondence: E-mails: godsonwu@126.com (Peide Wu); xxli@mail.sim.ac.cn (Xinxin Li)

Sample dimension calibration

The sample dimensions were determined using an optical microscope. As shown in Figure S1. The sample exhibits a diameter of 137 μm and a thickness of 37 μm. Accordingly, the volume is estimated to be approximately 3.64×10^{-7} cm^3, and the corresponding mass is about 0.418 μg.

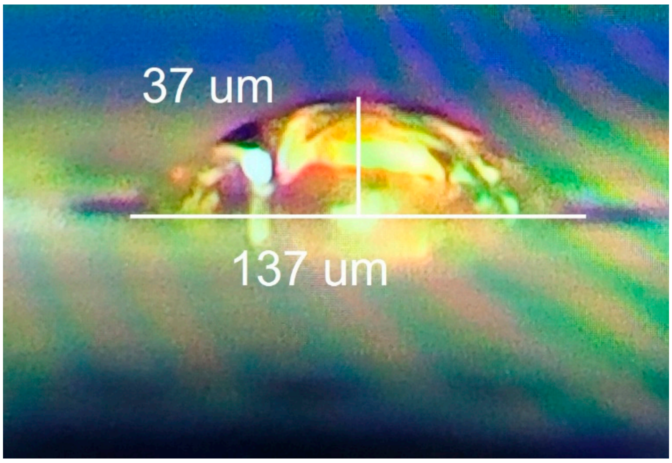

Figure S1. Optical images of the PA66-loaded sample

Chip fabrication process

The chip was fabricated on a (111) silicon wafer using MEMS technology. The detailed process flow is illustrated in Figure S2.

(a) Alignment marks were first defined on the silicon wafer by DRIE with an etching depth of 2 μm. A photoresist mask was then employed for phosphorus ion implantation in the n-type thermocouple region, with a dose of 8×10^{15} ion/cm^2 and an energy of 50 keV. After removing the photoresist, a second photolithography step was performed, followed by boron ion implantation in the p-type thermocouple region, with a dose of 1×10^{16} ion/cm^2 and the same energy of 50 keV. Subsequently, a 400 nm thick Low stress SiN_x layer and a 500 nm thick SiO_2 layer were deposited by LPCVD (low-pressure chemical vapor deposition).

(b) The 500 nm SiO_2 layer and the 400 nm SiN_x layer were sequentially etched by RIE to expose the single-crystal silicon. DRIE was then used to etch the exposed silicon to a depth of 4 μm, thereby forming the single-crystal silicon thermocouple legs.

(c) A 400 nm thick SiO_2 layer was deposited by LPCVD for sidewall protection. The wafer was then annealed at 1100 °C in an N_2 atmosphere for 300 min. After annealing, the junction depth reached 3 μm.

(d) Maskless anisotropic dry etching was carried out by RIE to remove the SiO_2 in the shallow trenches and expose the single-crystal silicon. Subsequently, DRIE was used to etch the bulk silicon to a depth of 40 μm, forming deep trenches for etching guidance. A 4 μm thick low-stress polysilicon layer was then deposited to seal the trenches. Chemical mechanical polishing (CMP) was performed to remove excess polysilicon from the surface. The polishing process was terminated once the dielectric layer was exposed, due to the combined chemical and mechanical action inherent to CMP.

(e) A 1 μm thick SiN_x supporting membrane was deposited by LPCVD to support the thermocouple leg structure. Contact holes between the metal and single-crystal silicon were then defined by photolithography, followed by sequential RIE of SiNx, SiO_2, and SiNx layers until the single-crystal silicon was exposed.

(f) A composite metal film was deposited by magnetron sputtering, consisting of 40 nm Cr, 100 nm Pt, and 3000 nm Au. After metal deposition, photolithography was used to pattern the metal interconnects and metal heating resistors. Ion Beam Etching (IBE) was then employed to remove the Cr/Pt/Au layers, completing the fabrication of the metal interconnections and heating resistors.

(g) A 500 nm thick SiO_2 layer was deposited by Plasma Enhanced Chemical Vapor Deposition (PECVD) as a passivation layer to prevent short circuits when the device is loaded with conductive materials. Release holes were then defined by photolithography, followed by RIE etching of the 500 nm SiO_2 and 1 μm SiN_x layers until the polysilicon was exposed.

(h) Finally, the release process was performed. Prior to release, the wafer was immersed in a low-concentration HF solution to remove the native oxide on the polysilicon surface within the release holes. Rapid release was then carried out using a TMAH solution with a concentration of 25 wt% at 80 °C.

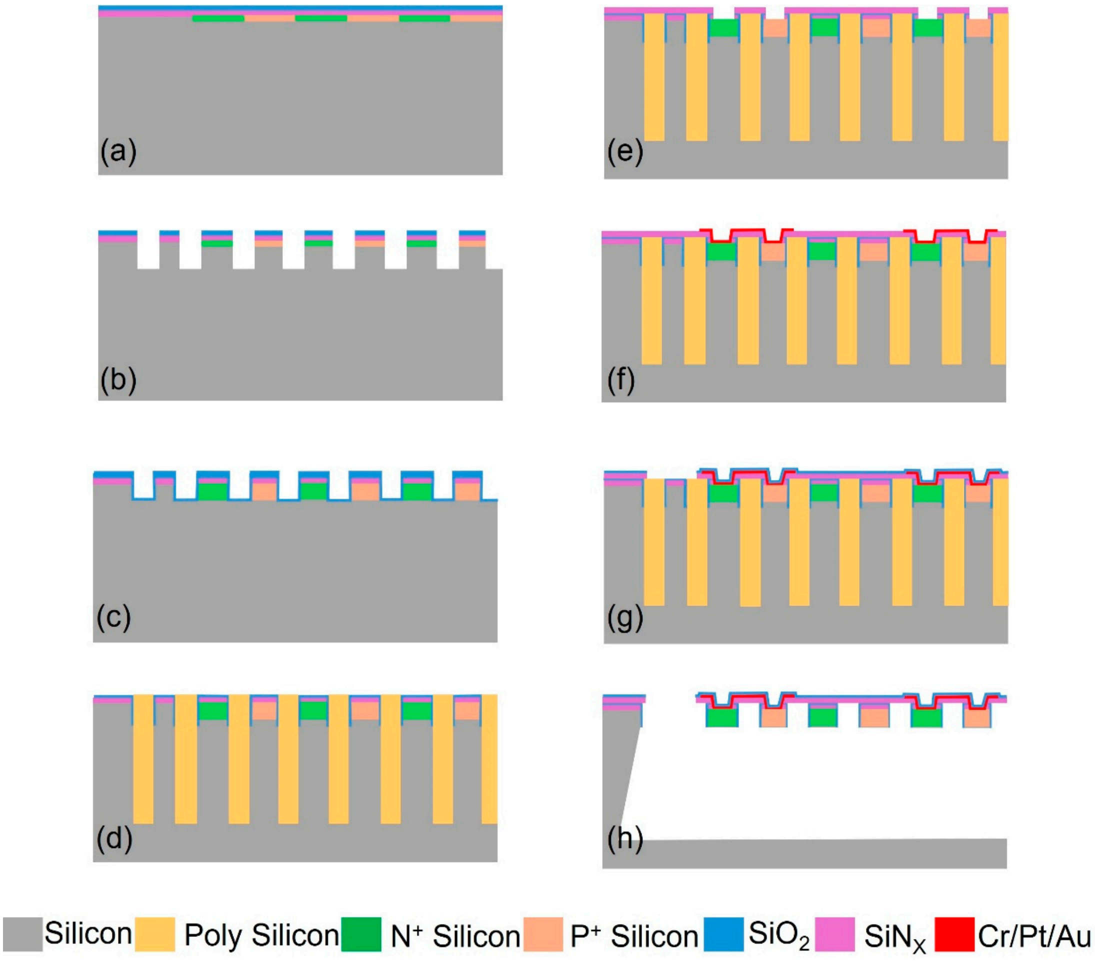

Figure S2. MEMS thermopile DSC chip fabrication process

Dimensional calibration of the MEMS thermopile DSC chip

Figure S3 presents the optical microscopy image of the MEMS thermopile chip. The overall chip dimensions are 1 mm in length and 2 mm in width. The suspended membrane region has a diameter of 640 μm, while the central heating area has a diameter of 240 μm. The heater exhibits an irregular polygonal geometry with a perimeter of approximately 1186.1 μm. It is composed of a Cr/Pt/Au composite metal layer structure, with individual thicknesses of 40 nm, 100 nm, and 3000 nm, respectively. In addition, a SiNx film with a thickness of 1 μm is deposited above the thermocouples. The thermal conductivity and heat capacity of this layer are 4 W/(m·K) and 7.72 × 10^{-7} J/K, respectively. In addition, the thermal conductivity at the interface between the film and the surrounding gas is 220 W/(m^2·K).

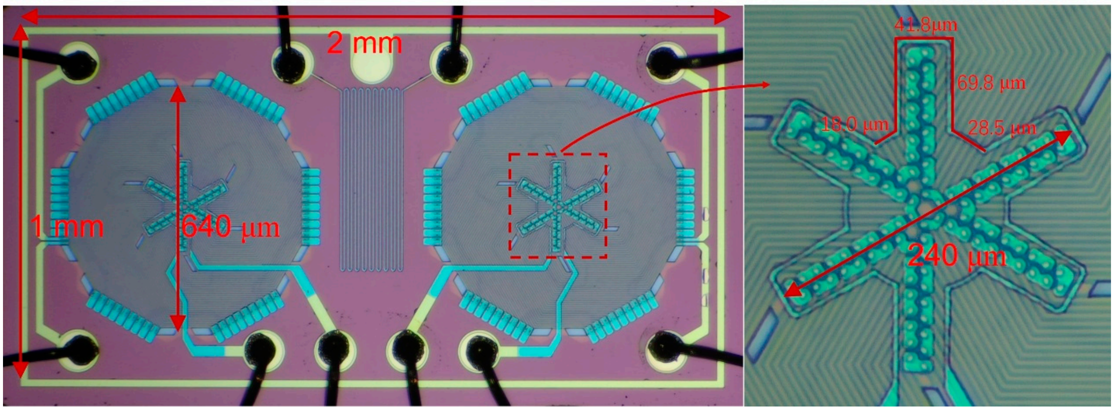

Figure S3. Optical microscopy image of the MEMS thermopile DSC chip

Table S1. Input parameters for the COMSOL finite element simulation.

Parameter	Value
Thickness of thermocouples (μm)	4
Width of thermocouples (μm)	3
Thickness of SiNx membrane (μm)	1
Height of isolation cavity (μm)	40
Thermal conductivity of Si (W/(m·K))	140
Thermal conductivity of SiNx (W/(m·K))	4
Thermal conductivity of metal (W/(m·K))	36

TCR of metal (1/K)	0.0016
Convection heat transfer coefficient (W/(m^2·K))	220
Boundary temperature (K)	293
Initial temperature (K)	293

Temperature gradient simulation

The COMSOL finite element simulation results, as shown in Figure S4, indicate that under heating voltages of 2 V, 3 V, and 4 V, the variation in the temperature gradient across the thermopile remains below 5%. This result confirms that the DSC chip exhibits excellent temperature uniformity.

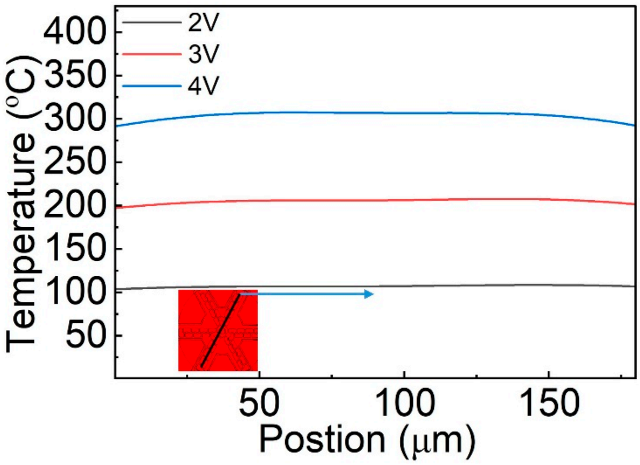

Figure S4. Temperature distribution maps at heating voltages of 2 V, 3 V, and 4 V

Step response of chip heating and cooling

To evaluate the dynamic thermal response performance of the MEMS thermopile DSC chip, step response measurements were conducted. As shown in Figure S5, the heating time constant and the maximum heating rate of the chip are 2.0 ms and 1.6 × 10^5 °C/s, respectively. The cooling time constant and the maximum cooling rate are 2.4 ms and 1.3 × 10^5 °C/s,

respectively.

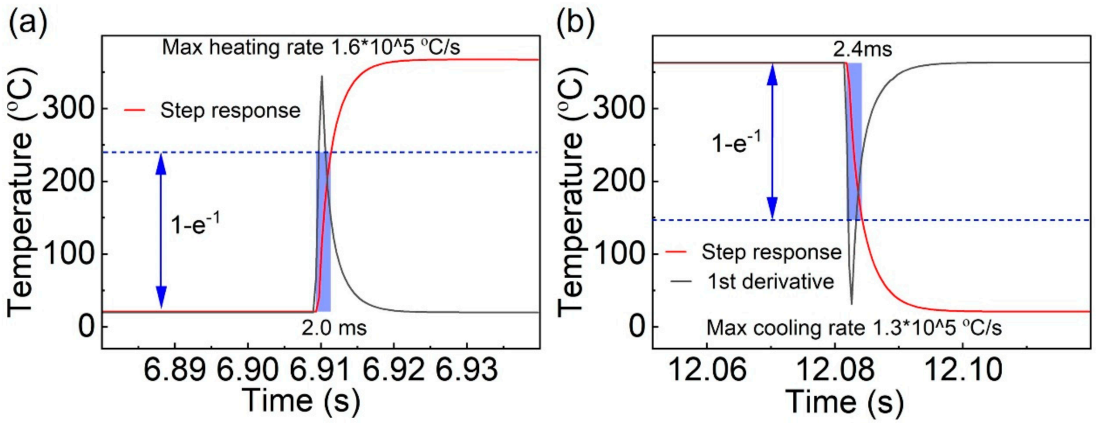

Figure S5. Step response of the MEMS thermopile DSC chip under unloaded conditions. (a) Heating step response curve; (b) Cooling step response curve

Table S2. Comparison of parameters between the MEMS thermopile DSC chip and the commercial chip UFS1

Parameter	Value MEMS thermopile DSC chip	Value UFS1[29]
Chip size (mm×mm)	2×1	1.6×1.6
Sample area (μm Ø)	240	500
Temperature response (mV/°C)	33.7	4
Power response (V/W)	140.2	24
Time constant for cooling of empty sensor (ms)	2.4	12
Time constant for heating of empty sensor (ms)	2.0	\

The comparison results indicate that the MEMS thermopile DSC chip exhibits higher temperature response and power response, as well as a smaller cooling time constant.

Characterization of Temperature Measurement Accuracy of the Chip-Based DSC

As shown in Figure S6, indium and tin were used to evaluate the temperature measurement accuracy of the MEMS thermopile DSC chip under scanning conditions. Measurements were conducted under an argon atmosphere at a heating rate of 100 °C/s, and the corresponding melting DSC curves were obtained. Analysis of the DSC curves yielded melting points of 156.52 °C for indium and 232.05 °C for tin, which are in excellent agreement with the standard values (156.51 °C and 231.89 °C). These results confirm the high accuracy of the chip under fast-scanning conditions.

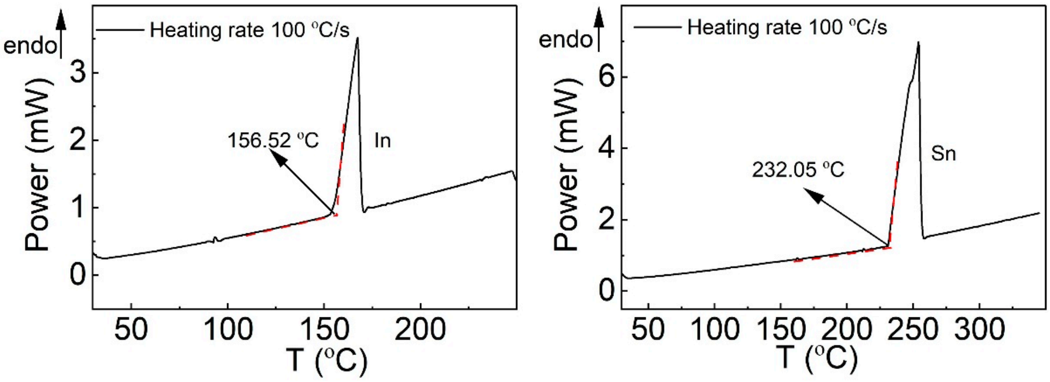

Figure S6. DSC melting curves of indium and tin measured using the MEMS thermopile DSC chip.

Determination of the quenching cooling rate of the MEMS thermopile DSC chip loaded with the sample

Figure S7. presents the temperature–time profiles of the chip loaded with a sample of approximately 0.418 μg during the quenching cooling process. As shown in Figure S7a, the time constant of the cooling process is determined to be 7.2 ms. The maximum achievable cooling rate reaches 8.4 × 10^4 °C/s, while the instantaneous cooling rate at 150 °C is approximately 1.03 × 10^4 °C/s. As illustrated in Figure S7b, the average cooling rate over the

temperature range from the test temperature down to 30 °C is evaluated to be 4177 °C/s.

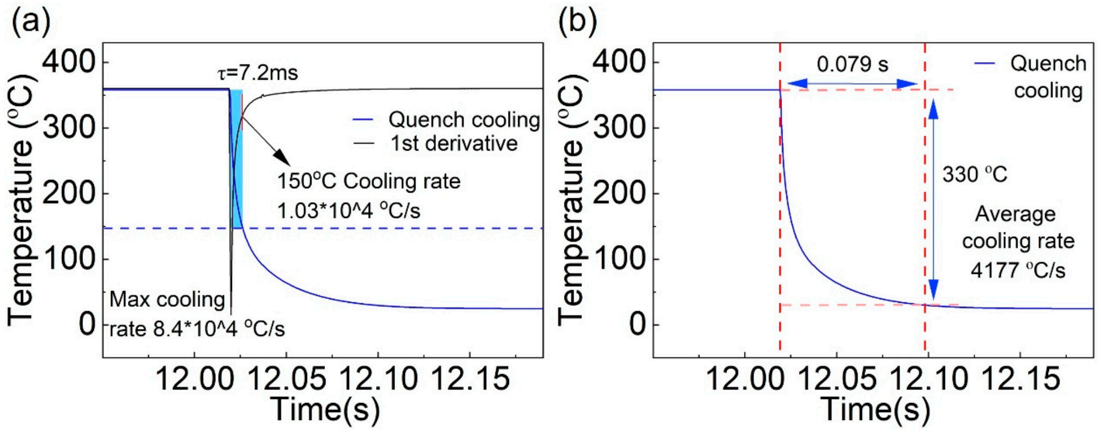

Figure S7. Quenching cooling rate measurement of the chip after loading the sample: (a) quenching cooling curve and its first derivative; (b) evaluation of the average cooling rate during the quenching process.

Infrared characterization of PA66 under different cooling conditions

The PA66 samples were first heated to 360 °C to completely eliminate thermal history, and subsequently cooled to room temperature at rates of 1 °C/s, 300 °C/s, and by quenching, respectively. Micro-infrared spectroscopy was employed to characterize the chemical structures of the samples obtained under different cooling conditions(Figure S8). The results indicate that the sample subjected to slow cooling at 1 °C/s exhibits characteristic absorption peaks at 936 cm^{-1} and 906 cm^{-1} in the infrared spectrum, which are assigned to the α crystalline form [45]. In contrast, the sample cooled at 300 °C/s shows a characteristic absorption peak at 1442 cm^{-1}, corresponding to the γ crystalline form[46,47]. Meanwhile, the quenched sample displays a fully amorphous state, with a characteristic absorption band appearing at 923 cm^{-1}[48].

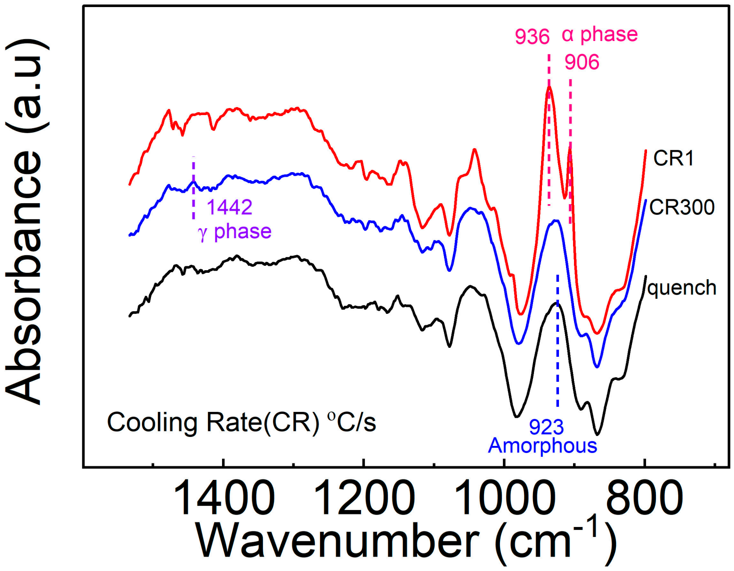

Figure S8. Infrared absorption spectra of samples subjected to different cooling conditions.

Comparison of homogeneous and heterogeneous nucleation cold crystallization under different heating rates

As previously demonstrated, homogeneous nucleation cold crystallization exhibits insensitivity to heating rates. We further investigate the relationship between heterogeneous nucleation cold crystallization behavior and heating rates. When the heating rate is slow, the heating process spans a significant time period, demanding extremely high baseline accuracy to prevent substantial errors. We examined the variation in heterogeneous nucleation and cold crystallization behavior at heating rates exceeding 10 °C/s. Our findings indicate that as the heating rate increases, heterogeneous nucleation and cold crystallization activity diminishes, becoming completely suppressed at 400°C/s (Figure S9).

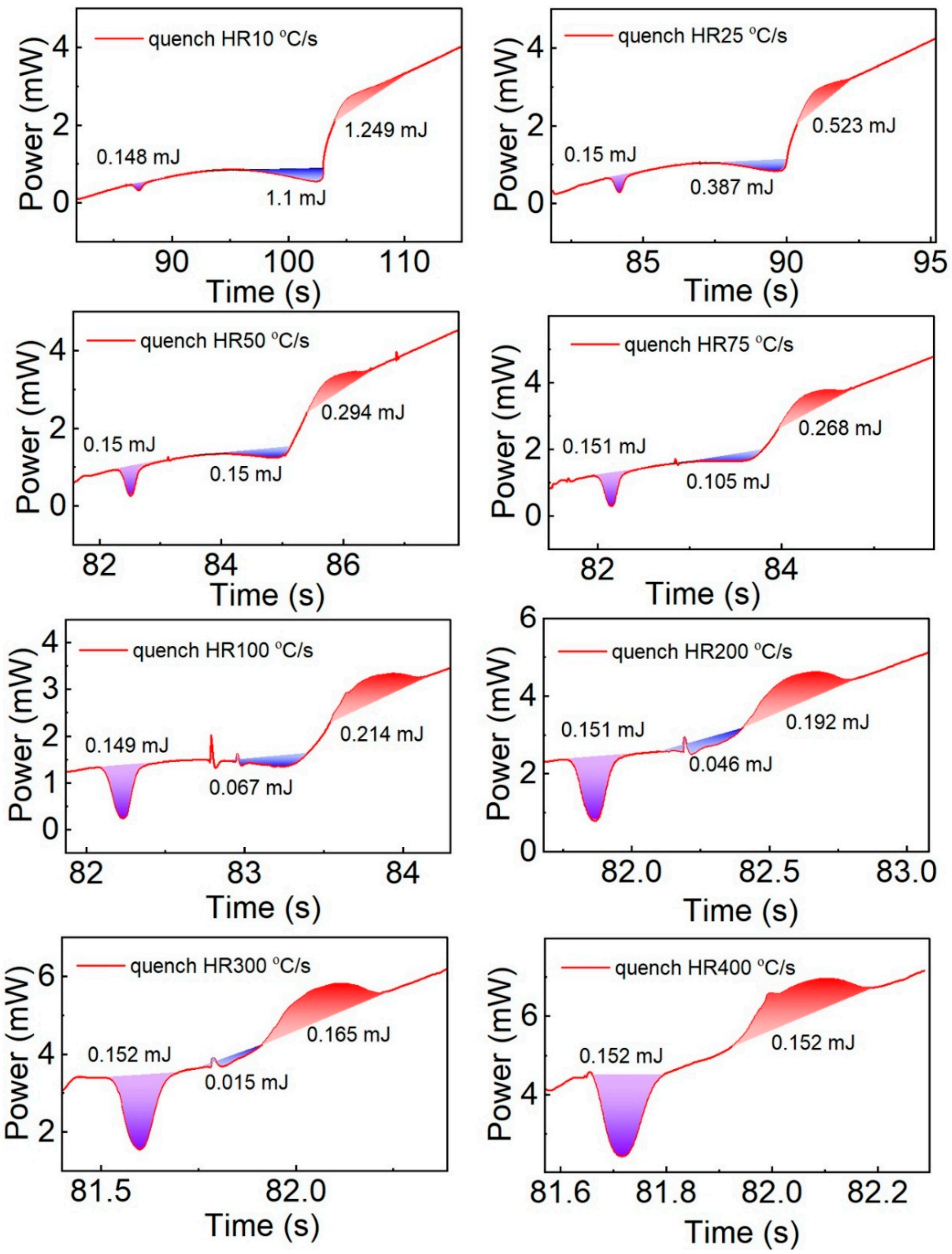

Figure S9. Integration analysis of heterogeneous nucleation cold crystallization enthalpy of quenched PA66 at different heating rates.
